# Coaching leadership and creative performance: A serial mediation model of psychological empowerment and constructive voice behavior

**DOI:** 10.3389/fpsyg.2023.1077594

**Published:** 2023-03-28

**Authors:** Chan Young Hwang, Seung-Wan Kang, Suk Bong Choi

**Affiliations:** ^1^College of Global Business, Korea University, Sejong City, Republic of Korea; ^2^College of Business, Gachon University, Seongnam, Republic of Korea

**Keywords:** coaching leadership, creative performance, psychological empowerment, constructive voice behavior, serial mediation analysis

## Abstract

This study empirically analyzes the role of coaching leadership in enhancing an organization’s creative performance, discussing and evaluating important mediating paths of coaching leadership regarding creative performance. As a result of an empirical analysis based on survey data collected from 332 employees of Korean companies, this study first confirms that coaching leadership has a positive effect on both employees’ creative performance. We also found that psychological empowerment and constructive voice behavior positively mediated the relationship between coaching leadership and creative performance. Finally, the serial mediating effect of coaching leadership on creative performance was tested through psychological empowerment and constructive voice behavior and confirmed to have a positive effect. This study indicates the importance of leadership as a critical variable that promotes employees’ creative performance. In addition, by confirming the serial mediating role of psychological empowerment and constructive voice behavior, this study improves understanding of key mechanism in which coaching leadership leads to creative performance.

## 1. Introduction

Today, organizations innovate to achieve sustained growth in response to rapid technological changes (Tierney and Farmer, 2011; Wang et al., 2021). Organizational innovation is important for ensuring that leaders and employees are aware of the flow of the market environment, respond actively to change, and exercise the necessary creativity (Amabile, 1988; Shalley, 1995; Shalley and Gilson, 2004). In other words, for a company to maintain continuous innovation, creative performance through various ideas must drive its success (Amabile, 1988; Shalley, 1995; Shalley and Gilson, 2004). Creative performance is the result of solving various problems that arise while performing tasks with creativity (Zhou and George, 2001; Choi et al., 2021). According to previous studies, the higher an individual’s internal motivation, the higher their creativity, organizational trust (Titrek, 2016), which promotes creative performance (Shalley and Gilson, 2004). The leader’s essential role is to promote creative performance by creating a positive atmosphere in the organization and giving employees internal motivation (Titrek and Çelik, 2011; Ye et al., 2020). In addition, to present directions of change and induce employees to exercise creativity, a leader’s role assignment and attention are needed (Abbas et al., 2022). From this point of view, this study analyzes the role of coaching leadership as an important factor in inducing creative performance. Coaching leadership focuses on growing and supporting organizational members (Whitmore, 2010) and refers to leaders’ actions that maintain positive relationships with organizational members and help them grow by drawing out their potential capabilities, unlike traditional leadership by direction and control around vertical hierarchies (Whitmore, 2010). Through coaching leadership, leaders set organizational goals, present future directions, and provide opportunities for competency development (Dahling et al., 2016; Cui et al., 2022). In other words, coaching leadership provides the resources needed to increase employees’ internal motivation, objectively evaluates the work process, and enables new ideas to be exercised through repeated feedback, leading to achieving organizational goals (Zhou and Shalley, 2003). This can lead to creative performance by employees voluntarily exerting the creativity necessary to achieve their organizational goals. Therefore, this study expects that coaching leadership is a factor in promoting creative performance and accordingly conducts an empirical analysis. On the other hand, psychological empowerment may promote creative performance by delegating and supporting employees to work with autonomy and confidence to achieve organizational goals (Zhang and Bartol, 2010; Malik et al., 2021). According to previous studies, coaching leadership promotes work autonomy and efficacy by communicating organizational goals and recognizing the meaning and importance of work by employees (Zhang and Bartol, 2010; Malik et al., 2021). Coaching leadership is expected to contribute to corporate performance by demonstrating creativity, as leaders stimulate employees’ passion, skills, and experience to achieve organizational goals (Gilson and Shalley, 2004; Yang et al., 2019). Therefore, this study analyzes how psychological empowerment mediates the relationship between coaching leadership and employee creative performance. In addition, employees are expected to improve creative performance by engaging in constructive voice behavior necessary for organizational innovation. According to previous studies, communication between leaders and employees promotes constructive voice behavior by relieving psychological pressure in job performance and increasing safety (Rusbult et al., 1988; Withey and Cooper, 1989), i.e., coaching leadership can increase confidence in performing tasks by supporting employees and providing them with the necessary opportunities for self-development (Eccles, 1993). This behavior is expected to contribute to creative performance by providing the constructive remarks employees need to achieve their goals and create various ideas (Edmondson et al., 2004). Therefore, this study analyzes whether constructive voice behavior mediates the relationship between coaching leadership and employees’ creative performance.

In addition, the relationship between creative performance and coaching leadership is expected to be promoted through psychological empowerment and constructive voice behavior, identifying a double mediating effect. This study explores the path through which coaching leadership promotes creative performance. In other words, coaching leadership promotes the constructive voice behavior necessary for innovative change by way of the internal motivation gained through employees’ psychological empowerment. Then, we analyze whether this constructive voice behavior increases creative performance based on the dual sequential mediating effect model.

## 2. Theoretical background and research hypotheses

### 2.1. Coaching leadership and creative performance

Coaching leadership is defined as the actions of leaders who support employee competency development and manage job performance to grow efficiently (Hamlin et al., 2009; Hagen and Aguilar, 2012). Therefore, coaching leadership can be seen as a leader’s behavior that improves the relationship between leaders and employees by supporting employee growth and managing job performance to achieve corporate goals. Specifically, coaching leadership presents employees with four components: direction, development, accountability, and relationship based on interviews (Stowell, 1986). On the other hand, creative performance is defined as the challenges employees need to achieve organizational goals by presenting potential ideas and deriving innovative solutions (Zhou and George, 2001; Choi et al., 2021). In other words, creative performance means the results of solving problems with various approaches and new ideas that maintain sustainable growth by increasing the competitiveness of the organization. This study infers based on the following logic that coaching leadership will positively affect employees’ creative performance.

First, leaders and employees establish directions and goals for company growth through the communication and diverse sense making processes (Lussier and Achua, 2004; Schilling et al., 2022). In this process, leader’s behavior is typically closely monitored and interpreted by their follower (Schilling et al., 2022). Coaching leadership can increase communication, improving employees’ understanding of the organization’s future direction and internal motivation by recognizing their roles within the organization (Shalley and Gilson, 2004). Through coaching leadership, internal motivation increases, and the remaining employees can voluntarily present ideas and actively work (Mulki et al., 2006). Therefore, in promoting organizational innovation, coaching leadership can lead to creative results by presenting more diverse ideas and applying them to work (Zhou, 2003; Shalley and Gilson, 2004). Second, coaching leadership provides not only material support but also mental support (Baron and Morin, 2010; Yuan et al., 2019), offering the resources needed to realize employees’ various ideas toward promoting organizational change (Srivastava et al., 2006). Coaching leadership can improve employees’ psychological expectation that they will receive the resources needed to achieve their goals and actively perform their tasks (Yuan et al., 2019). Therefore, coaching leadership promotes creative performance by allowing employees to present various ideas through material and mental support.

Third, coaching leadership involves evaluating employees’ work processes and providing appropriate feedback. Through this process, employees can effectively perform by recognizing and supplementing errors in the work process (Raza et al., 2017). In addition, as the feedback process is repeated, satisfaction with the organization increases by recognizing the leader as part of a cooperative relationship (Mulki et al., 2006). This process generates the various ideas necessary to achieve organizational goals by increasing positive emotions regarding the relationship between leaders and employees (Fredrickson, 2001; Choi et al., 2015). Therefore, employees can present the necessary ideas after receiving feedback on problems that arise in the process of organizational change, leading to creative performance. As such, coaching leadership can generate creative results by increasing employees’ understanding of organizational goals through active communication and by providing various ideas and problem-solving methods alongside necessary resources and feedback (Lussier and Achua, 2004). Therefore, the following hypothesis was established.

**Hypothesis 1 (H1).** Coaching leadership will have a positive (+) effect on creative performance.

### 2.2. Psychological empowerment as a mediator

Psychological empowerment refers to the psychological state of having influence in the organization by leaders stimulating employees’ skills and experiences (Eccles, 1993) and employees recognizing the meaning of the assigned tasks (Thomas and Velthouse, 1990; Corsun and Enz, 1999). In this study, psychological empowerment is expected to play a mediating role in the relationship between coaching leadership and employees’ creative performance. In other words, it is inferred that employees will have a positive effect on psychological empowerment and, as a result, a positive effect on creative performance through coaching leadership. First, according to previous studies, coaching leadership involves presenting a company’s future vision and providing a guide for employees to understand the meaning of performance tasks (Stowell, 1986). This strengthens internal motivation by instilling an awareness that the work assigned in the innovation process is important (Amabile, 1988). Coaching leadership contributes to creative performance by recognizing the authority and role granted to employees to achieve corporate goals and promoting the psychological internal motivation to engage in tasks (Spreitzer, 1995), i.e., by actively presenting the various ideas needed to solve problems (Redmond et al., 1993).

Second, coaching leadership provides employees with opportunities for systematic competency development to enhance their competitiveness (Dahling et al., 2016; Cui et al., 2022). This effectively increases confidence by developing the competence employees need for their work (Eden, 1992; Kovjanic et al., 2013; Chen et al., 2014). Therefore, as employees increase their capabilities and strengthen confidence in their assigned tasks, they will actively present various ideas and creative problem-solving methods to contribute to creative outcomes (Bandura, 1977; Edmondson et al., 2004; Wang et al., 2021). Third, coaching leadership involves creating an organizational culture based on interpersonal skills to form a positive relationship with the organization (Gilley et al., 2010; Cui et al., 2022). The more positive the organization’s atmosphere, the higher the employees’ cooperative feelings (Cui et al., 2022). As a result of these strengthened cooperative feelings, employees will contribute to creative performance by presenting various ideas necessary for the organizational change process (Baumeister and Leary, 1995; Wang et al., 2022). In this way, coaching leadership increases employees’ voluntary internal motivation by improving their understanding of the company’s future vision and supporting capacity development (Dahling et al., 2016; Cui et al., 2022), therefore helping them actively demonstrate creativity during organizational change. Therefore, in this study, the following hypothesis is established.

**Hypothesis 2 (H2).** Psychological empowerment positively mediates the relationship between coaching leadership and creative performance.

### 2.3. Constructive voice behavior as a mediator

Constructive voice behavior refers to employees’ voluntary expression of positive opinions needed for organizational innovation (Organ and Ryan, 1995; Morrison, 2011), which means that they actively communicate individual beliefs needed for organizational change as a result of the positive relationship created between leaders and employees. In this study, constructive voice behavior is expected to mediate the relationship between coaching leadership and creative performance. In other words, it is inferred that coaching leadership will have a positive effect on employees’ constructive voice behavior, and as a result, it will also have a positive effect on creative performance.

First, coaching leadership provides mental support for employees to be confident even if they face unexpected work failures or risks (Baron and Morin, 2010; Yuan et al., 2019). Coaching leadership allows employees to actively express their beliefs by increasing employees’ psychological safety even if they face unexpected confrontations while proceeding with organizational change (Srivastava et al., 2006). Therefore, to lead organizational change successfully and constructively, various opinions must be sufficiently expressed. These active employee voice behaviors contribute to organizational performance by creating new ideas and problem-solving methods (Edmondson et al., 2004). Second, according to previous studies, coaching leadership helps to form a culture of free communication between leaders and employees (Fredrickson, 2001; Choi et al., 2015), improving understanding of others’ opinions through communication, relieving psychological pressure, and increasing safety (Cropanzano and Mitchell, 2005). It also leads to active publishing behavior by providing the necessary speaking opportunities for the organizational change process (Avey et al., 2012; Zhou and Long, 2012). Therefore, a healthy organizational communication culture formed by coaching leadership that promotes employees’ opportunities to speak can lead to creative performance by creating various ideas necessary for organizational change (Edmondson et al., 2004).

Third, coaching leadership includes providing effective and psychological comfort in building positive relationships with employees by offering feedback and promoting interaction with them (Owens et al., 2015; Yang et al., 2021). In addition, the interaction between leaders and employees promotes the constructive voice behavior needed to achieve organizational goals while raising trust (Fredrickson, 2001; Choi et al., 2015). Therefore, coaching leadership contributes to creative performance by actively promoting new ideas, encouraging interaction with leaders for enhanced psychological comfort, and fostering the constructive voices necessary for organizational change (Edmondson et al., 2004). In this way, coaching leadership builds positive relationships through interactions between leaders and employees (Owens et al., 2015; Yang et al., 2021), and these relationships contribute to creative performance by leading employees to voluntarily engage in the constructive voice behavior necessary to achieve organizational goals (Edmondson et al., 2004). Therefore, for this study, the following hypothesis is established.

**Hypothesis 3 (H3).** Constructive voice behavior positively mediates between coaching leadership and creative performance.

### 2.4. Integrated model: A serial mediating effect

In considering the mediating effects proposed in this study, the sequential double mediating effects can be estimated. In other words, it can be inferred that psychological empowerment and constructive voice behavior will double mediate the relationship between coaching leadership and creative performance. First, coaching leadership stimulates internal motivation by presenting guides to increase understanding of the organization’s future vision and the meaning of tasks, therefore allowing employees to recognize the importance of tasks assigned to them (Zhou and George, 2001; Choi et al., 2021). This interaction increases employees’ psychological empowerment and leads to constructive voice behavior without adding or subtracting the individual’s conviction needed for organizational change (Cropanzano and Mitchell, 2005). Employees’ constructive statements to enhance organizational performance can be seen as promoting creative performance by creating new solutions (Zhou and George, 2001; Choi et al., 2021). Second, coaching leadership helps employees develop job skills and stimulates personal growth by forming a positive relationship between leaders and employees (Stowell, 1986). These actions encourage close relationships with leaders, and employees enhance their sense of authority and ownership psychologically, which results in increased constructive voice behavior where employees express various opinions regarding organizational development (Baumeister and Leary, 1995; Wang et al., 2022). As such, constructive voice behavior for the purpose of organizational change contributes to creative performance by providing various ideas about existing work methods and processes, complementing deficiencies, and deriving new alternatives (Edmondson et al., 2004).

In this way, coaching leadership promotes constructive voice behavior, where employees express the individual beliefs necessary for organizational change and development without hesitation, by increasing psychological empowerment through interaction between leaders and employees (Owens et al., 2015; Yang et al., 2021). Employees’ constructive voices lead to improved creative performance as they develop creative solutions (Edmondson et al., 2004). Therefore, in this study, the following hypothesis is established.

**Hypothesis 4 (H4).** Psychological empowerment and constructive voice behavior double mediate coaching leadership and creative performance.

**FIGURE 1:**
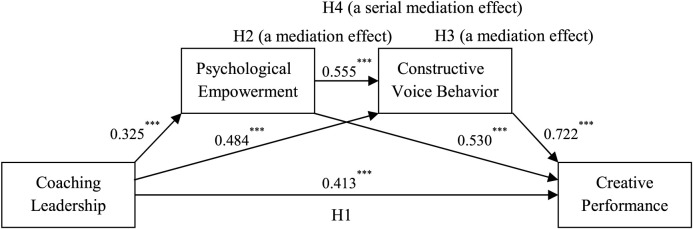
Hypothesized research model. *N* = 321. ***p < 0.001 (two-tailed test).

The hypothesized research model is presented in Figure 1.

## 3. Methodology

### 3.1. Sample and procedure

Data collection for this study was conducted through an online and offline questionnaire in Korean manufacturing firms for the month of February 2022. The respondent’s main tasks were managerial, clerical, production, sales and customer service. We visited each company, explained the intent of the survey to the department managers, and received their permission. Of these 340 questionnaires, 19 questionnaires were considered invalid due to omitted or incorrect answers. Eventually, 321 responses were used for data analysis. The demographic characteristics of the respondents are as follows. Among the 321 respondents of this study, 160 (49.8%) were male, and 161 (50.2%) were female. The average age of the respondents was 45.1 years (SD = 10.06), with 6.9% of them averaging between 20 and 29 years, 26.8% averaging between 30 and 39 years, 29.3% averaging between 40 and 49 years, 30.5% averaging between 50 and 59 years, and 6.5% averaging over 51 years old. Regarding education background, 17.8% had completed high school, 14.3% had college diplomas, 55.5% held bachelor’s degrees, 9.0% had master’s degrees, and 1.6% held doctorates. The average organizational tenure was 11.02 years (SD = 8.14).

### 3.2. Measures

The original questionnaire was in English, then it was translated into Korean using the Brislin (1980) back-translation procedure. A professional translator translated the original version into Korean, then a bilingual scholar (who neither was aware of the study’s purpose nor had seen the original survey) back-translated it into English. As commonly used in previous studies (e.g., Ko and Yu, 2017; Chegini et al., 2020 for coaching leadership; Zhang and Bartol, 2010 for psychological empowerment; Yang et al., 2019; Avey et al., 2012 for constructive behavior; Ahmad et al., 2019 for creative performance; Shin et al., 2019), all instruments used in this study were aggregated into one dimension and used for empirical analysis.

First, we used Stowell’s (1986) 13-item scale to measure coaching leadership. Respondents rated items on five-point Likert scales that ranged from 1 (strongly disagree) to 5 (strongly agree). Sample items are: “My supervisor actively helps me set specific work goals for myself,” and “My supervisor gives me a clear direction for my work.” The Cronbach’s alpha was 0.946. Next, we used Spreitzer’s (1995) 12-item scale to measure psychological empowerment. Respondents rated items on five-point Likert scales that ranged from 1 (strongly disagree) to 5 (strongly agree). Sample items are: “The work I am doing is very important to me,” and “Activities related to my job are personally meaningful to me.” The Cronbach’s alpha was 0.901. Then, we used Van Dyne and LePine (1998) 12-item scale to measure constructive voice behavior. Respondents rated items on five-point Likert scales that ranged from 1 (strongly disagree) to 5 (strongly agree). Sample items are: “I can make constructive suggestions to my supervisor on issues that affect the organization,” and “I encourage my colleagues to take a joint interest in improving problems that affect the organization.” The Cronbach’s alpha was 0.901. Lastly, we used Oldham and Cummings’s (1996) and Morrison and Phelps’s (1999) six-item scale to measure creative performance. Respondents rated items on five-point Likert scales that ranged from 1 (strongly disagree) to 5 (strongly agree). Sample items are: “I come up with creative ideas when I do my work,” and “I am always on the lookout for new technology, process or product ideas.” The Cronbach’s alpha was 0.845.

## 4. Results

### 4.1. Correlation and reliability analysis

A correlation analysis was conducted by using SPSS 21.0 to investigate the relationships among the measured variables. Means, standard deviations, and correlations among the variables are reported in Table 1. Coaching leadership was significantly related to creative performance (*r* = 0.415, *p* < 0.01), psychological empowerment (*r* = 0.328, *p* < 0.01), and constructive voice behavior (*r* = 0.487, *p* < 0.01).

**TABLE 1:** Descriptive statistics and correlations.

Variable	Mean	SD	1	2	3	4	5	6	7	8	9
1. Gender (0 = M;1 = F)^a^	1.502	0.501									
2. Age	45.137	10.064	−0.015								
3. Education^b^	4.396	1.302	−0.090	-0.078							
4. Tenure^c^	11.019	8.141	−0.145**	0.474**	0.153**						
5. Position	2.461	1.227	−0.291**	0.289**	0.318**	0.500**					
6. Coaching leadership	3.234	0.773	−0.022	-0.024	-0.017	0.027	0.023	(0.946)			
7. Psychological empowerment	3.559	0.587	−0.040	0.130*	0.065	0.190**	0.188**	0.328**	(0.901)		
8. Constructive voice behavior	3.437	0.593	−0.013	0.062	0.007	0.106	0.141*	0.487**	0.657**	(0.826)	
9. Creative performance	3.308	0.612	−0.110*	0.109	0.086	0.135*	0.219**	0.415**	0.628**	0.765**	(0.845)

*N* = 321; *p < 0.05; **p < 0.01; Reliability alpha (α) coefficients are reported in diagonal.

^a^M = male; F = female.

^b^Education = years of formal education.

^c^Tenure = number of years.

### 4.2. Common method bias and confirmatory factor analysis

To assess common method variance, we followed the recommendation by Podsakoff et al. (2003) and conducted Harman’s single-factor test by loading all the items of the study constructs into an exploratory factor analysis. The results indicate that no single factor explains more than 38.61% of the covariance among the variables. We also conducted a confirmatory factor analysis (CFA) on the measures of the key variables by using AMOS 20.0 to verify their factor structure and construct validity. We modeled four factors: coaching leadership, psychological empowerment, constructive voice behavior, and creative performance. The results are shown in Table 2. This theoretical four-factor model provided a reasonable fit to the data (χ^2^ = 1,134.511), degree of freedom (df) = 616, Comparative Fit Index (CFI) = 0.923, Tucker-Lewis Index (TLI) = 0.917, Root Mean Square Residual (RMR) = 0.046, Root Mean Square Error of Approximation (RMSEA = 0.051). Additionally, a series of Chi-square difference tests revealed that the four-factor model fits the data significantly better than several alternative measurement models (Table 2). The results verify the theoretical four-factor model, thus supporting discriminant validity among the measures.

**TABLE 2:** A confirmatory factor analysis for comparison of measurement models.

Model	χ^2^ (df)	CFI	TLI	RMR	RMSEA	Δχ^2^ (df)
Theoretical 4-factor model	1,134.511 (616)	0.923	0.917	0.046	0.051	
3-factor model^a^	1,352.867 (619)	0.901	0.883	0.053	0.061	218.356 (3)***
2-factor model^b^	2,155.726 (621)	0.773	0.756	0.106	0.088	802.859 (2)***
1-factor model^c^	2,876.673 (622)	0.666	0.642	0.103	0.106	720.947 (1)***

*n* = 321. ***p < 0.001 (two-tailed test).

^a^Three-factor model with psychological empowerment and constructive voice behavior on the same factor.

^b^Two-factor model with coaching leadership and psychological empowerment, constructive voice behavior, and creative performance on the same factor.

^c^One-factor model with coaching leadership, psychological empowerment, constructive voice behavior, and creative performance on the same factor.

χ^2^, chi-square; df, degrees of freedom; CFI, comparative Fit Index; TLI, Tucker-Lewis Index; RMR, root mean square residual; RMSEA, root mean square error of approximation.

The index for the overall confirmatory factor analysis is presented in Table 2. The results of the confirmatory factor analysis were χ^2^ (df) = 1,134.511(616), Incremental Fit Index (IFI) = 0.924, TLI = 0.917, CFI = 0.923, Goodness-of-Fit Index (GFI) = 0.832, RMR = 0.046, RMSEA = 0.051, and Normed Fit Index (NFI) = 0.847. As shown in Table 2, although GFI and NFI are not in the best model indicator, χ2 (df), IFI, TLI, CFI, and RMSEA are included in the best model indicator, and the values of RMR are at acceptable levels (Hair et al., 2010). Therefore, the results of confirmatory factor analysis are acceptable. In addition, the average variance extracted (AVE), the variance size that explains the concept of the measurement variables, is greater than 0.5, the reference value. The construct reliability value exceeded 0.7, thus supporting the convergent validity and reliability of the constructs (Fornell and Larcker, 1981).

### 4.3. Hypothesis testing

We used the hierarchical regression analysis by using SPSS 21.0 to test the hypothesized relationships (Wiklund and Shepherd, 2005; Hoch et al., 2010). The results of the hierarchical regression analysis are shown in Table 3. H1 proposed that coaching leadership is positively related to creative performance. After controlling for gender, age, education, organization tenure, and interaction frequency, Table 3 shows the positive relationship between coaching leadership and creativity performance (β = 0.413, *p* < 0.001; Model 2), therefore supporting H1. H2 posited that psychological empowerment would mediate the relationship between coaching leadership and creative performance such that coaching leadership would enhance psychological empowerment and psychological empowerment would lead to better creative performance. In bootstrapping to test the mediating effect of coaching leadership on creative performance through psychological empowerment, the observed coefficient effect was 0.0568, and the 95% bias-corrected bootstrap confidence interval (CI) did not include 0 (0.0252, 0.0987) (see Table 4). Thus, we consider H2 supported. H3 posited that constructive voice behavior would mediate the relationship between coaching leadership and creative performance such that coaching leadership would enhance constructive voice behavior and constructive voice behavior would lead to better creative performance. In bootstrapping to test the mediating effect of coaching leadership on creative performance through constructive voice behavior, the observed coefficient effect was 0.1432, and the 95% bias-corrected bootstrap confidence interval (CI) did not include 0 (0.0919, 0.1994). Thus, we consider H3 supported. H4 predicted that psychological empowerment and constructive voice behavior would serially mediate the relationship between coaching leadership and creative performance such that coaching leadership would enhance psychological empowerment, which would in turn increase constructive voice behavior, and then increased constructive voice behavior would enhance creative performance. Indirect testing with bootstrapping produced a coefficient of 0.0860, and the 95% bias-corrected bootstrap CI also excluded zero (0.0494, 0.1308). These results indicate that H4 is supported.

**TABLE 3:** Results from hierarchical linear regression analysis with creative performance and coaching leadership.

Variables	Creative performance			
	**Model 1**	**Model 2**	**Model 3**	**Model 4**
Gender	-0.055	-0.050	-0.059	-0.077*
Age	0.057	0.080	0.046	0.064
Education	0.030	0.045	0.031	0.063
Tenure	0.009	-0.009	-0.058	-0.029
Position	0.172*	0.162**	0.102	0.069
Coaching Leadership		0.413***	0.241***	0.064
Psychological Empowerment			0.530***	
Constructive Voice Behavior				0.722***
R^2^	0.054	0.224	0.461	0.611
ΔR^2^		0.170	0.238	0.149

*n* = 321. *p < 0.05; **p < 0.01; ***p < 0.001 (two-tailed test). Standardized regression coefficients reported.

**TABLE 4:** Bootstrapped indirect effects.

Indirect effects	Effect	Boot SE	Boot LLCI	Boot ULCI
CL → EM → CP	0.0568	0.0188	0.0252	0.0987
CL → CVB → CP	0.1432	0.0277	0.0919	0.1994
CL → EM → CVB → CP	0.0860	0.0206	0.0494	0.1308

CL, coaching leadership; EM, psychological empowerment; CVB, constructive voice behavior; CP, creative performance.

## 5. Conclusion and implications

To accommodate rapid environmental changes and maintain a stable business, it is important to find new ideas and problem-solving methods for producing creative results (Gilson and Shalley, 2004; Shalley et al., 2009). However, despite this need, research on the coaching leadership as a factor in increasing creative performance is still insufficient (Kim et al., 2013; Wang et al., 2017; Peng et al., 2019). Therefore, this study analyzes leadership’s effect on employees’ creative performance as well as the mediating role and a serial mediating effect of psychological empowerment and constructive voice behavior in these relationships.

### 5.1. Theoretical implications

This study’s results provide the following theoretical implications. First, many studies have examined the effectiveness of leadership as a prerequisite for organizational creative performance (Shalley et al., 2009). However, research linking coaching leadership to creative performance is still insufficient. This study confirms the important role of coaching leadership in promoting creative performance. Coaching leadership is a leader’s behavior that contributes to creative performance by supporting employee growth and job competency (Stowell, 1986; Baron and Morin, 2010; Yuan et al., 2019). This study expands research on leadership and creativity by revealing that coaching leadership is an important determinant of creative activities and performance, and it helps employees accurately recognize changes in the organizational environment and supports necessary competency development.

Second, this study analyzes both psychological and behavioral situations to explain the process by which coaching leadership promotes creative performance. Psychological empowerment was selected as a parameter representing a psychological situation, and as a result, it was confirmed as an effective parameter affecting creative performance by increasing employees’ psychological empowerment through linkage action (Hagen and Aguilar, 2012). In addition, constructive voice behavior was selected as a parameter representing behavioral situations, and as a result, employees’ voice behavior had a positive effect on creative performance (Organ and Ryan, 1995; Morrison, 2011). This study provides insights to understand coaching leadership by analyzing its mediating effect on creative performance through employees’ psychological and behavioral situations. Third, to explain how coaching leadership promotes creative performance, this study analyzes the sequential double mediating effect through psychological empowerment and constructive voice behavior. In previous studies, the relationship between leadership and innovation performance has been analyzed for direct or primary indirect effects. However, this study provides a different analysis model by analyzing the double mediating effect of coaching leadership on creative performance through psychological and behavioral situations. Therefore, it provides insights into understanding the path from coaching leadership to creative performance.

### 5.2. Practical implications

This study has the following practical implications based on its empirical results. First, as a result of this study’s empirical analysis, it was found that coaching leadership positively contributes to creative performance. This effect contributes to the increase in creative performance through close supervisory load interactions, with coaching leadership providing material and psychological support to employees (Stowell, 1986; Redmond et al., 1993; Baron and Morin, 2010; Yuan et al., 2019). Therefore, an organization must be aware of the positive effects of leadership and establish systematic selection, and education and training programs to develop and strengthen managers’ coaching leadership capabilities. More specifically, we suggest that organization establish effective selection system based on coaching instruments to recruit managers who already possess coaching capability externally and promote employees who are potential coaches internally (Cui et al., 2022; Wang et al., 2022). In addition, organizations also could provide or support formal training and education program to help managers become effective coaches. For instance, organizations could use external professional coaching institutes to help managers become certified coaches (Cui et al., 2022). In general, we also found that leaders are required to prepare proper leadership styles and strategies for developing management skills to increase the efficiency of their organizations (Fenitra et al., 2022). Second, organization should facilitate the coaching organizational culture, motivating the leader to have various kinds of coaching behavior (Wang et al., 2022). For instance, organization could support managers’ coaching related behavior such as guidance, facilitation and inspiration of coaching activities which further enhance the employee’s sense of belonging to the organization and of psychological safety so that employees can continue their creative work.

Third, this study confirms that psychological empowerment and constructive voice behavior had an important mediating effect on connecting and promoting creative outcomes not only individually but also together. Therefore, to improve an organization’s creative performance, leaders need to develop coaching leadership, as well as establish an open organizational culture that guarantees free communication and support systems at various organizational levels to strengthen employees’ psychological empowerment and constructive voice behavior.

Finally, the sample of this study found that the average age and tenure of employees were relatively higher. It implies that organizations that would like to increase their creative performance might benefit from employees who are old and more long tenured. This is because they might benefit from providing incentives and support for performing their job creatively (Kim et al., 2015). Such organizations might also encourage these employees to develop high-quality relationship and cooperation with their leaders, which may make them feel more empowered, produce more taking charge behavior, and result in better creative performance (Marinova et al., 2010). This result suggests that it may be more effective to encourage longer tenured employees instead of shorter tenured employees to engage in creative behavior. Perhaps, longer tenured employees will exhibit greater performance benefits when engaging in performing creative tasks. In addition, older and long tenured employees may be less threatened by a failure in the course of performing their job creatively, and they also can take better position to utilize required organizational resources for creative performance. In this vein, the present study demonstrates that employees with relatively old age and longer organizational tenure may be better able to translate the benefits of coaching leadership and psychological empowerment into creative performance. Therefore, we suggest that organization may consider carefully the positive role of old and long tenured employees for enhancing creative performance.

### 5.3. Limitations and directions for future research

Despite the useful implications, this study also has the following limitations, so it is expected to be reflected in future studies. First, this study collected data utilizing a self-report questionnaire method, so there may be a problem of convenience in the same method (Spector, 2006). Therefore, in future studies, it is necessary to increase the reliability of the research results by diversifying the response time and the response sources. Second, this study employed a cross-sectional research method by collecting data through a questionnaire at a specific point in time. However, because the results may vary depending on the employee’s psychological state and environmental factors, it is expected that future research based on longitudinal data will help increase the reliability of the results and reveal the causal relationship. Third, this study used an analysis model at the individual level to analyze the relationship between coaching leadership and creative performance, but it did not consider all variables at the team and organizational levels. However, employees’ creative activities and performances are influenced by variables such as team atmosphere, innovative organizational culture, and team cohesion. Therefore, in future studies, more useful implications can be derived if a multiple-level analysis includes influencing variables at the team or organization level. Finally, although the data used in this study came from multiple job areas of Korean manufacturing industry, this study was conducted on samples drawn exclusively from South Korean workers in manufacturing industry. Therefore, future research should extend to other industry and other emerging and developed countries for useful implications and generalizability of research results.

## Data availability statement

The raw data supporting the conclusions of this article will be made available by the authors, without undue reservation.

## Author contributions

CYH prepared the first draft of the manuscript. SBC supervised the study and refined the draft into a publishable manuscript. CYH and SBC collected the data and performed empirical analysis. In addition to motivating the publication of this manuscript, S-WK added valuable theoretical and methodological insights based on his knowledge and expertise regarding the topic. All authors read and agreed to the submitted version of the manuscript.
